# Barriers to ART adherence in sub-Saharan Africa: a scoping review toward achieving UNAIDS 95-95-95 targets

**DOI:** 10.3389/fpubh.2025.1609743

**Published:** 2025-06-10

**Authors:** Judie Magura, Sibongile R. Nhari, Thokozani I. Nzimakwe

**Affiliations:** ^1^Africa Health Research Institute (AHRI), Durban, South Africa; ^2^School of Laboratory Medicine and Medical Sciences, University of KwaZulu-Natal, Durban, South Africa; ^3^School of Management, Information Technology and Governance, University of KwaZulu-Natal, Durban, South Africa

**Keywords:** ART adherence, 95-95-95 UNAIDS, HIV/AIDS, adult men and women, global HIV funding

## Abstract

**Introduction:**

With the 2025 UNAIDS 95-95-95 deadline upon us, significant gaps remain in achieving universal HIV care and treatment targets, particularly in Sub-Saharan Africa. Despite years of intensified global efforts, progress has lagged, partly driven by the multifaceted challenges of non-adherence to ART, shaped by social, economic, structural, and individual factors. These challenges have been compounded by the ever-changing landscape of global HIV funding, further undermining treatment outcomes. Addressing these interconnected barriers is essential to identify and implement targeted, evidence-based solutions.

**Methods:**

To explore these challenges and potential interventions, a scoping review was conducted, searching through PubMed and Dimensions databases for peer-reviewed articles published from 2020 through February 2025. Eligible studies focused on barriers to ART adherence in adult men and women ≥ 18 years old living with HIV, aligning with the UNAIDS 95-95-95 framework in sub-Saharan Africa.

**Results:**

Of the 4,928 articles screened, 21 were included in this scoping review. Although the search period was extended to February 2025, no eligible studies published in 2025 were identified. Barriers to ART adherence were multifaceted, spanning individual-level issues such as mental health issues and substance abuse; social barriers including stigma and intimate partner violence; and economic factors, including food insecurity, transport costs, and income instability. Structural barriers such as health system fragmentation, clinic accessibility, and drug stockouts were also common and often worsened by the COVID-19 pandemic, which disrupted service delivery and exacerbated socioeconomic vulnerabilities. While only peer-reviewed articles were included in the analysis, recent UNAIDS reports and reputable media sources, such as The Guardian, were referenced to contextualize the emerging impact of the 2025 HIV funding cuts, which have not yet been reflected in the academic literature.

**Conclusion:**

Our findings emphasize the urgent need for targeted, multi-level interventions to address persistent economic, social, psychological, and policy barriers to ART adherence. A sustainable funding framework, combined with financial support, mental health services, and community-based care models, is crucial for improving retention and long-term adherence. These insights are essential for shaping policies, strengthening HIV service delivery, and sustaining momentum toward the 95-95-95 targets amid systematic challenges.

## 1 Introduction

The UNAIDS 95-95-95 targets reflect a global commitment to ending the HIV/AIDS epidemic by ensuring that 95% of all people living with HIV (PLHIV) know their status, 95% of those diagnosed receive sustained antiretroviral therapy (ART), and 95% of those on ART achieve viral suppression (1). These targets were established on the understanding that they are essential in reducing HIV-related morbidity, mortality, and transmission, with the ultimate aim to eliminate AIDS as a public health threat by 2030 (2). However, progress in sub-Saharan Africa remains vulnerable. As of 2023, the Eastern and Southern Africa region, home to the highest burden of HIV globally, had reached 93% of people knowing their status, 83% of those diagnosed receiving ART, and only 78% of those on ART achieving viral suppression, with wide disparities by age and gender (3). While these figures reflect meaningful progress, gaps remain, and persistent barriers such as stigma, gender-based violence, socio-economic disparities, and healthcare access limitations continue to undermine adherence and retention in care (4, 5). These long-standing challenges have been compounded by recent disruptions, including the residual effects of the COVID-19 pandemic and global HIV funding cuts announced in 2025, which threaten to stall or reverse progress.

Achieving the 95-95-95 goals requires the active participation of both men and women, whose ART adherence is critical not only for individual viral suppression and reduced risk but also for the broader sustainability of public health gains. In sub-Saharan Africa, where the burden of HIV remains high and ART scale-up has been one of the region’s most significant health achievements, maintaining high levels of adherence among adults is essential to preserve these gains (6). Adult men and women are central to treatment adherence efforts, both as individuals managing their health and as caregivers ensuring the well-being of children and adolescents affected by HIV. Therefore, understanding factors that influence their ART adherence is essential to designing effective, context-responsive interventions in a rapidly evolving landscape.

This scoping review synthesizes recent evidence (2020–early 2025) on the barriers to ART adherence among adults and examines how these challenges impede progress toward achieving the UNAIDS 95-95-95 targets. Analyzing studies conducted over the past five years provides a comprehensive overview of longstanding and emerging adherence challenges specific to sub-Saharan Africa, highlighting regional disparities and offering evidence-based recommendations to strengthen ART adherence strategies. By focusing on post-2020 studies, this review captures both persistent challenges and newer disruptions linked to the COVID-19 pandemic and recent funding shifts, contextual factors that have compounded existing gaps. In doing so, it fills a critical gap in the literature and offers timely insights to inform targeted, context-responsive strategies for improving treatment retention and health outcomes for individuals and communities affected by HIV.

### 1.1 Defining adherence

Various definitions have shaped our understanding of poor ART adherence, each offering unique insights into patient behaviors and treatment challenges. Self-reported measures are commonly used, and they capture adherence patterns based on whether individuals are currently taking ART, have missed doses in the past seven days, or have discontinued treatment within the previous year (7, 8). Some studies considered in this review assessed adherence difficulties using the AIDS Clinical Trials Group (ACTG) questionnaire (9), which provides a subjective yet valuable perspective on patient experiences.

Beyond self-reports, electronic monitoring methods offer an objective measure of adherence. For instance, the Wisepill pillbox system records a missed dose when the device is not opened within a two-hour window of the prescribed time, allowing for real-time tracking (10). Pill count methods assess adherence by determining the proportion of returned ART tablets, with a threshold of less than 95% considered indicative of non-adherence (7, 11). Pharmacy refill records offer another lens, where missed ART refills, re-initiation of first-line ART, or switching to second-line therapy indicate potential adherence challenges (12). Additionally, viral load monitoring serves as a critical biological marker of adherence, with virological failure often defined as a viral load exceeding 20 copies/mL, while a higher threshold of ≥1,000 copies/mL signals significant treatment failure. Lastly, general adherence levels provide an overarching assessment, with adherence considered suboptimal when less than 90% of prescribed doses are taken. These diverse definitions are summarized in Table 1, reflecting a multidimensional understanding of adherence.

**Table 1:** Definitions and measurement methods of ART adherence in included studies.

Method	Description	Indicators of poor adherence	Referenced studies
Self-report	Patient-reported adherence, including current ART use, missed doses (e.g., past 7 days), or treatment discontinuation in the past year.	Any missed doses, treatment interruption, or non-initiation	Twimukye et al. (8) and Ngowi et al. (7)
ACTG questionnaire	Structured questionnaire capturing subjective adherence behaviors and experiences.	Patient reports of missed doses or difficulties with adherence	Jones et al. (9)
Electronic monitoring	Devices like Wisepill record real-time pillbox openings within a specific time window.	No device opening within a 2-h window of the prescribed dose	Wagner et al. (10)
Pill count	Calculation based on returned pills compared to expected usage.	<95% of prescribed doses taken	Mubiana-Mbewe et al. (11) and Ngowi et al. (7)
Pharmacy refill records	Tracking ART pickup and refill patterns, including missed refills or treatment switches.	Missed refills, re-initiation, or switch to second-line ART	Jennings Mayo-Wilson et al. (12)
Viral load monitoring	Biological indicator of adherence, based on plasma HIV RNA levels.	>20 copies/mL = virological failure; ≥1,000 copies/mL = treatment failure	Standard clinical thresholds
General thresholds	The overall adherence percentage is based on various methods.	<90% of prescribed doses taken	General benchmark across studies

While definitions and thresholds vary, the common thread across is that poor ART adherence is a failure to consistently take medication as prescribed, whether through missed doses, delayed refills, or inconsistent clinic attendance that undermines viral suppression. For this review, we define ART adherence as the consistent and timeous intake of prescribed antiretroviral medication, ensuring optimal viral suppression, which can be measured through self-reports, electronic monitoring, pill counts, viral load assessments, and pharmacy refill records.

## 2 Methodology

This study was conducted as a scoping review in accordance with the framework developed by Arksey and O’Malley (13) and further refined by Levac, Colquhoun, and O’Brien (14). The methodology adheres to the PRISMA-ScR (Preferred Reporting Items for Systematic Reviews and Meta-Analyses Extension for Scoping Reviews) guidelines to ensure transparency and replicability. Peer-reviewed studies published from 2020 through February 2025, focusing on barriers to ART adherence in adults (≥18 years) living with HIV, aligning with the UNAIDS 95-95-95 framework, were screened for inclusion. The studies that met this criterion and were included in the final analysis are summarized in Supplementary Table S1.

In keeping with the scoping review methodology, no formal assessment of the quality of bias was conducted, as the aim was to map the scope and nature of available evidence rather than evaluate methodological rigor (14). However, we prioritized peer-reviewed literature and included only studies with clearly described methods to ensure baseline level of credibility.

**Figure 1:**
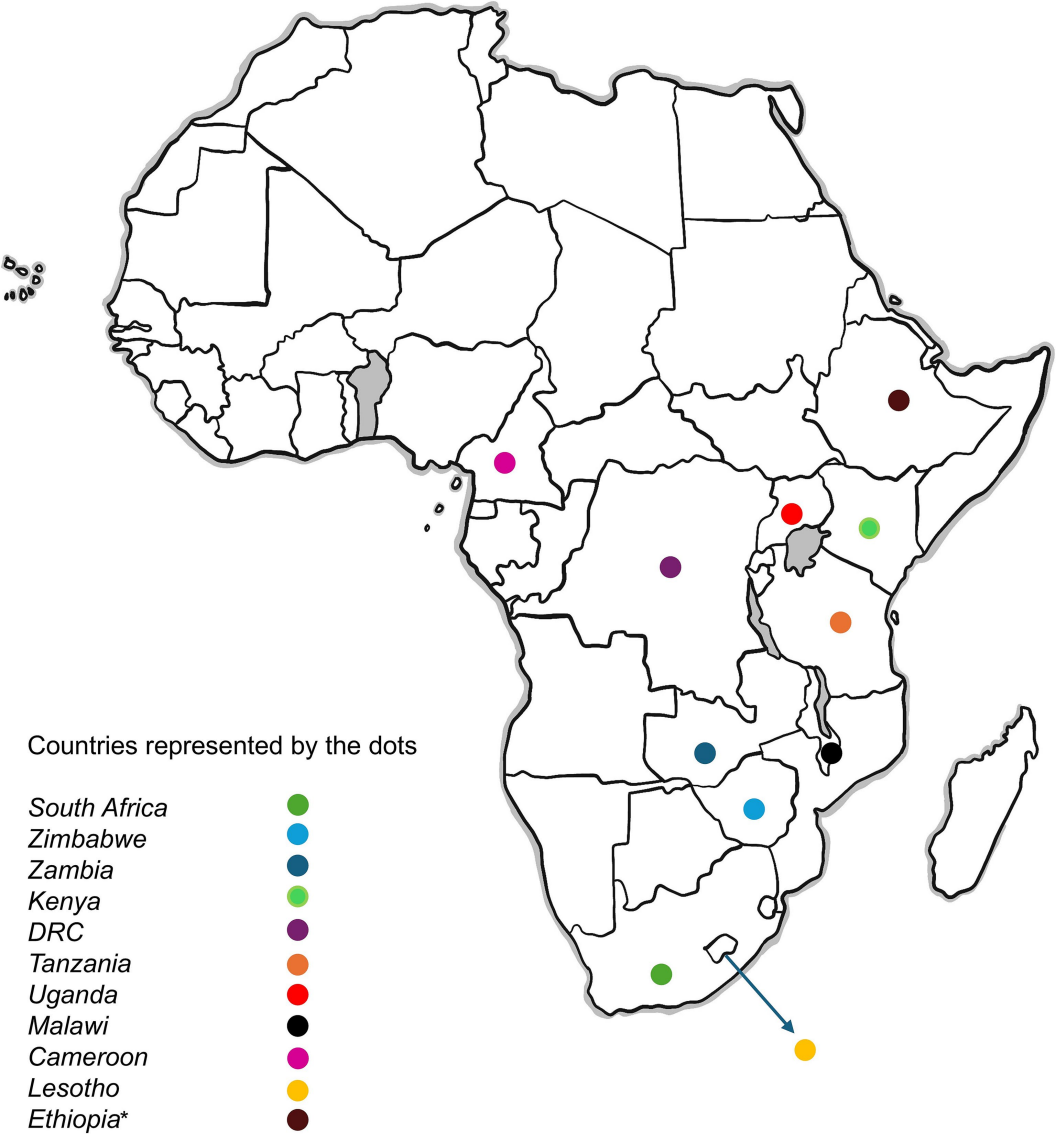
Map showing Sub-Saharan African countries included in the scoping review. The dots represent the countries featured in the review. *Ethiopia is included as an additional country due to significant disruptions to HIV services associated with the 2025 U.S. foreign aid freeze, as reported by UNAIDS and reputable media sources. Original image designed by Rocketpixel/Freepik (freepik.com).

The selected studies primarily consisted of clinical trials, employing quantitative or mixed methods approaches, with some incorporating qualitative components. To provide a regional perspective, sub-Saharan Africa was considered as countries in the region are perceived to experience similar challenges without much disparity, influencing and impacting each other. These countries, generally classified as low- and middle-income, are represented in the studies included in this review and depicted in Figure 1. Only studies published in English were included. To augment peer-reviewed literature, UNAIDS reports and credible media sources were consulted to contextualize findings and highlight emerging and real-time issues that have yet to be widely documented in academic publications. Studies focusing solely on ART efficacy without discussing adherence challenges were excluded. Further exclusions included conference abstracts, reviews, opinion pieces, and grey literature lacking empirical data, as well as studies unrelated to the UNAIDS 90–90-90 or 95-95-95 framework.

### 2.1 Data sources and search strategy

**Figure 2:**
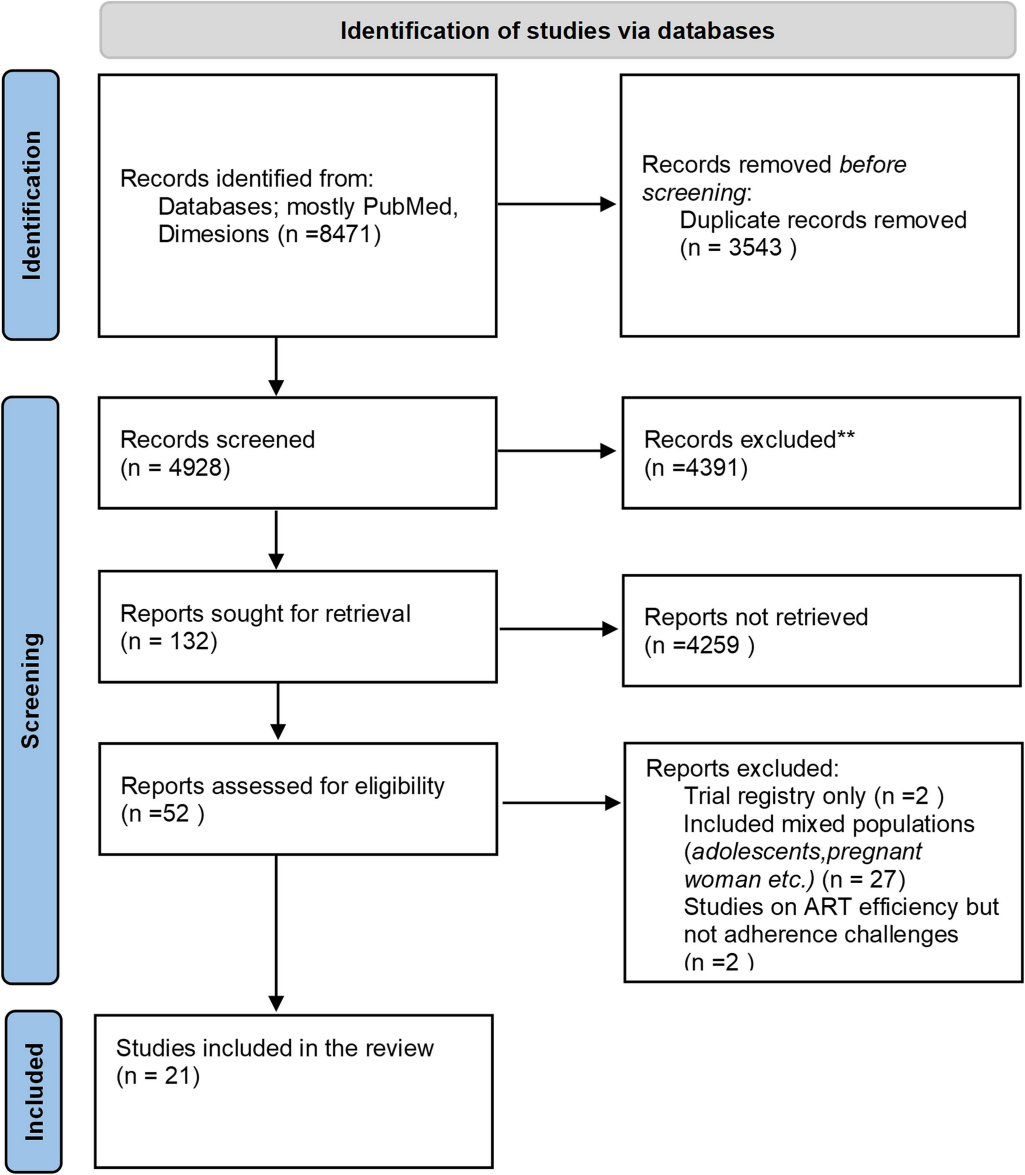
Preferred reporting items for systematic reviews and meta-analyses (PRISMA) flow diagram illustrating the identification, screening, and inclusion of studies in the review.

A broad literature search was initially conducted across multiple databases, including PubMed, Dimensions, Google Scholar, JSTOR, and Web of Science. However, most eligible articles were ultimately identified through PubMed and Dimensions, while other databases yielded minimal or no additional eligible studies. As a result, only articles from PubMed and Dimensions were included in the final analysis. Please refer to Figure 2 for an overview of the study selection process.

The database search strategy was conducted using combinations of key terms grouped around three main concepts: (1) ART adherence (e.g., *“Treatment adherence in HIV/AIDS” or “ART adherence”*), (2) barriers of influencing factors (e.g., *“Factors affecting ART adherence” or “Barriers* to ART adherence*”*), and context-specific terms (e.g.*, “UNAIDS 95-95-95” and “Africa” OR “sub-Saharan Africa”*). These term sets were searched separately or in smaller combinations using Boolean operators (AND/OR) where appropriate.

All references from this search were imported into Ryyan’s systematic review management software, where duplicates were first removed. The study selection process involved a two-phase screening approach: title and abstract screening by both researchers to assess relevance, followed by full-text review of articles meeting inclusion criteria for final selection. Data extraction focused on capturing key details, including study characteristics [author(s), year, country, study design] and participant characteristics (age and gender), alongside types of barriers identified (structural, economic, behavioral, psychological) and their impact on ART adherence and UNAIDS targets.

## 3 Ethical considerations

As this study is a review of existing literature, it does not require ethical approval. However, all included studies were assessed for adherence to universal and contextual ethical research standards.

## 4 Discussion

Several interconnected factors have been identified across the sources as negatively impacting ART adherence, including individual, social, economic, and structural barriers, along with funding challenges. These are further complicated by a complex interplay between the factors, all contributing to disparities in achieving the UNAIDS 95-95-95 targets. Despite significant advancements in HIV treatment, barriers to adherence persist, undermining efforts to achieve viral suppression and improve health outcomes among PLHIV (15, 16). This section synthesizes recent findings on the key challenges affecting ART adherence, focusing on adult males and females in sub-Saharan Africa.

### 4.1 Individual factors

Multiple individual factors significantly affect adherence to ART. Studies included here discuss how depression significantly impairs ART adherence, with symptoms such as loss of interest and poor concentration reducing motivation for self-care behaviors, including taking medication and attending clinic visits (10). In sub-Saharan Africa, depression among PLHIV has been recorded to range from 9 to 32% (16). Mpungu further identifies depression, anxiety, and post-traumatic stress disorder (PTSD) as key barriers to adherence, as they disrupt daily routines and diminish motivation to maintain treatment. A study in Uganda found a bidirectional relationship between depression and ART adherence, where worsening depression predicted lower adherence, and vice versa (10). Similarly, during the COVID-19 lockdown, individuals experiencing heightened depression reported lower adherence scores (15). Depression not only reduces motivation but also impairs cognitive function, memory, and concentration, making it more difficult to adhere to a consistent medication schedule.

Beyond mental health, substance use, particularly alcohol consumption, has been consistently identified as a significant barrier to ART adherence. Evidence from studies conducted in Kenya, Uganda, and South Africa suggests that alcohol and drug use impair decision-making and disrupt daily routines, leading to poor engagement, lower retention in care, missed doses, and increased virological failure rates (9, 17–19). Being male has also been identified as a risk factor for adherence challenges, as men report lower self-efficacy, higher rates of pill count non-adherence, and greater treatment discontinuation. In addition, perceptions of health status influence adherence for both men and women. Many individuals delay ART initiation if they feel healthy, highlighting the need for continuous adherence education (8). Furthermore, self-efficacy plays a crucial role in sustaining treatment; patients with high confidence in their ability to manage ART are more likely to adhere, while those with low confidence are at greater risk of non-adherence (20). Knowledge and awareness also impact adherence, as misinformation, such as the belief that ART is unnecessary when symptoms are absent, contributes to inconsistent medication use (20).

Addressing these individual factors through mental health support, behavioral counseling, and targeted interventions can improve ART adherence and contribute to achieving the UNAIDS 95-95-95 targets.

### 4.2 Social factors

Social factors significantly play a critical role in adherence to ART. Intimate partner violence (IPV) has been identified as one of the strongest predictors of non-adherence, often exacerbated by alcohol use (21). This is supported by findings that experiencing all forms of violence was associated with lower ART adherence scores (15). Gender dynamics have often been reported to shape these challenges, with men’s unhealthy alcohol use contributing to violence against their female partners, consequently hindering women’s ability to adhere to their medication (17, 21). Healthcare workers and female patients have reported male partners preventing women from accessing ART (22).

In addition to the impact of IPV, other social factors significantly influence ART adherence. The stigma associated with HIV remains a major barrier, affecting men and women differently. In the global south, masculine gender norms emphasize self-reliance, discouraging help-seeking behaviors and leading men to anticipate stigma, which in turn hinders testing, care-seeking, and adherence (8). Conversely, women face gender-specific challenges, including higher risks of IPV and stigma upon HIV status disclosure, subsequently further complicating adherence (23).

Beyond gender differences, stigma manifests in multiple forms, such as community stigma, internalized stigma, household-level stigma, and stigma within healthcare settings. This can lead to fear of disclosure, hiding medication, low self-esteem, and ultimately, poor adherence (9). Internalized stigma refers to self-directed shame and negative beliefs that PLHIV absorb from societal attitudes. It is often associated with secrecy, diminished self-worth, and reluctance to access care or adhere to treatment. In South Africa and Uganda, internalized stigma has been linked to delayed ART initiation and reduced treatment motivation, especially among women who fear judgement even in supportive clinical environments (9, 20, 24). Household-level stigma, characterized by gossip, blame, and insults related to HIV status, is associated with decreased adherence (19). HIV-related stigma within healthcare facilities is another potent deterrent to adherence. Many people living with HIV in the Global South fear judgment, breaches of confidentiality, or differential treatment when they visit clinics. Studies consistently show that stigma whether over discrimination or anticipated shame, discourages patients from engaging fully in care (25). Patients have reported overhearing nurses gossiping about them or being exposed by open clinic layouts that compromise privacy (26). Similarly, research in African contexts finds that enacted stigma by healthcare workers (e.g., scolding or breaching confidentiality) and the fear of such stigma make patients more likely to skip visits or even stop ART to avoid embarrassment (25, 27). A recent review on stigma in young HIV patients confirmed stigma is a “significant barrier” to ART adherence and retention in care, as it contributes to non-disclosure and psychosocial stress (25). In short, when patients feel stigmatized or unsafe in healthcare environments, their adherence suffers, and they may miss doses, hide their medication, or drop out of treatment entirely.

### 4.3 Economic and structural factors

Economic and structural barriers to ART adherence frequently overlap, as individual financial constraints, such as poverty, food insecurity, and transport costs, are often exacerbated by systematic issues like poor infrastructure, limited clinic availability, and healthcare system inefficiencies. This intersection creates layered obstacles that affect both the ability to access care and maintain adherence.

#### 4.3.1 Poverty, food insecurity, and prioritization of basic needs

Socioeconomic challenges are consistently highlighted across the studies as significant barriers to ART adherence (23, 28–30). Studies in sub-Saharan Africa, including South Africa and Uganda, have shown that PLHIV in low-income households face multiple livelihood-related barriers. Food insecurity, in particular, contributes to inconsistent ART use, as side effects can be aggravated when medication is taken without food. As noted in the Gumede et al. (20) study in a rural South African community, low household income was associated with suboptimal pill count adherence and food insecurity was linked to virological failure in men. Participants in the Mcinziba et al. (19) study also reported prioritizing basic needs like food over ART-related costs.

#### 4.3.2 Employment constraints

Employment-related challenges also significantly impact ART adherence, though men and women experience them differently. For men, logistical barriers such as inflexible work schedules, travel for employment, and lack of clinic flexibility increase the risk of treatment interruption (8). Many are employed in informal sectors, where missing work for clinic visits can mean the loss of income, making it difficult to prioritize ART refills over survival needs (19). Men were also more likely than women to highlight work-related challenges as obstacles to seeking and receiving care. For women, economic burdens linked to caregiving responsibilities, such as childcare, household duties, and school fees, often take precedence over personal healthcare, further complicating adherence (23). Young adults also reported competing work schedules as a reason for forgetfulness, emphasizing the need for adherence support (8).

#### 4.3.3 Transport and geographic barriers

While ART may be free, the cost and logistics of transportation remain substantial barriers. For instance, a study conducted in South Africa found that long travel distances and associated financial costs were significant barriers to ART adherence, where approximately 63.6% of respondents traveled more than 15 km to access treatment centers. In addition, 65% of these respondents reported missing their ART medication doses more than once per week (31). Similarly, research in Kampala showed that individuals frequently missed appointments because they could not afford transportation, and long travel times contributed to treatment interruptions (32).

In Malawi, distance-related barriers to ART adherence are worsened by seasonal factors, especially during the rainy season when flooding disrupts access to various facilities, including healthcare. Mphande et al. (41) point out that severe flooding destroys and damages infrastructure, creating hazardous travel conditions that lead to treatment interruptions for individuals living with HIV. Furthermore, the destruction caused by flooding often results in losing personal belongings, including medications, further hindering adherence. These challenges undergird the critical need for resilient healthcare delivery systems with the potential to withstand climatic adversities and ensure continuous ART access.

#### 4.3.4 Health system limitations

Clinic accessibility is a critical structural factor influencing ART adherence, particularly in low-resource communities and environments where healthcare infrastructure is often inadequate. Limited clinic operating hours, typically restricted to weekday daytime, pose a major obstacle for patients who work or have family obligations. Qualitative evidence from South Africa found that inflexible 7 am–4 pm clinic hours routinely clashed with patients’ jobs and home responsibilities, forcing many to choose between earning an income and managing their health (33). Such scheduling conflicts often lead to missed visits, delayed medication refills, treatment interruptions, and eventual disengagement from care (33). Similar findings across African contexts noted that inconvenient clinic times contribute to non-adherence, especially among employed individuals who cannot easily get time off work (34). In addition to inconvenient hours, overburdened clinics and staff shortages result in prolonged wait times, which discourage patients from staying engaged in treatment. In sub-Saharan Africa, public clinics have been reported to be so congested that patients spend several hours waiting for brief consultations or medication refills, imposing opportunity costs such as loss of income or neglected family responsibilities (27). These frustrations, compounded over time erode patients’ motivation and lead to missed visits, treatment interruptions, and ultimately suboptimal adherence (27). Reducing wait times and improving scheduling flexibility are therefore critical to strengthening ART adherence and sustaining long-term engagement.

Despite persistent economic and structural, several strategies have been tested to improve ART adherence in resource-limited settings. Strong social support, particularly from partners and within households, has been shown to improve ART adherence, emphasizing the need for peer support programs and household-level interventions (29, 30). Financial incentives have proven to be successful in alleviating this burden, helping improve ART adherence by covering transport costs (28). Decentralized ART distribution models, such as community-based and mobile ART delivery models, reduce travel burden and improve treatment outcomes (35). These efforts are important in contexts shaped by systemic and geographical marginalization. As recent studies suggest, adherence support must also address income instability and the daily trade-offs people face in managing their health (19). Scaling up these flexible, patient-centered approaches remains essential for sustaining engagement in care.

### 4.4 Drug stockouts

Stockouts of antiretroviral drugs in clinics lead directly to treatment interruptions. In many low-income countries, supply chain issues or funding gaps cause ART medications to be unavailable at times. When patients arrive for refills during a stockout, they may receive an incomplete supply or none at all, forcing them to miss doses. A qualitative study in Uganda highlighted the ripple effects of ART stockouts: patients were given fewer pills and had to return more frequently, incurring extra travel costs and even risking disclosure of their HIV status to employers due to repeated absences (27). Such disruptions strain patients and undermine consistent pill-taking. A 2020 review noted that drug stock-outs remain a significant structural barrier to adherence in sub-Saharan Africa (34). Even short gaps in medication can lead to viral rebound and drug resistance, so stockouts seriously jeopardize treatment success. Unfortunately, during crises like the COVID-19 pandemic, stockout risks have been amplified in some regions, further threatening continuous ART use (27). Ensuring an uninterrupted drug supply is fundamental to supporting adherence.

### 4.5 The evolving global HIV funding-current USA presidency administration’s impact

The recent U.S. foreign aid freeze has dealt a devastating blow to global HIV programs, threatening decades of hard-won progress toward epidemic control. On January 20, 2025, during his first day in office, President Donald Trump launched his second term with an executive action that froze approximately 90% of the U.S. Agency for International Development’s (USAID) foreign aid contracts. This abrupt halt in funding has had a profound impact on HIV/AIDS programs in Southern Africa, a region heavily reliant on U.S. support for ART scale-up, viral load monitoring, and treatment adherence counseling. While specific figures detailing the exact amount withdrawn from Southern Africa’s HIV/AIDS initiatives are not readily available, the overall reduction in funding is substantial and has led to significant disruptions in healthcare services across the region. Experts warn that these cuts could result in up to 500,000 additional deaths in South Africa over the next decade if disruptions persist (42).

The scope of the impact has been staggering. For instance, the Elizabeth Glaser Pediatric AIDS Foundation (EGPAF) was forced to close programs in Lesotho, Eswatini, and Tanzania, affecting HIV treatment for approximately 350,000 individuals, including nearly 10,000 children dependent on ART continuity and support services (42). Clinics have closed, frontline staff have been laid off, and supply chains for essential diagnostics and medications have been disrupted.

These developments mark a continuation of policy trends set during Trump’s first term (2017–2021), when the Mexico City Policy, also known as the Global Gag Rule, was reinstated and expanded to apply to all U.S. global health funding (36, 37). This policy barred funding to foreign NGOs that provided, promoted, or even discussed abortion services, even with non-U.S. funds. Although its stated goal was reproductive health control, the expansion had severe unintended consequences for HIV service integration, community outreach, and Sexual and Reproductive Health and Rights (SRHR)-linked adherence programs, particularly in sub-Saharan Africa. In contrast, President Joe Biden revoked the Gag Rule in January 2021, signaling renewed support for the integrated SRHR and HIV services. His administration broadly endorsed the President’s Emergency Plan for AIDS Relief (PEPFAR) and multilateral global health engagement. However, in 2023, instead of the typical multi-year reauthorization, Congress extended PEPFAR only through March 2025, introducing uncertainty about the program’s long-term future (38, 39). With this temporary extension and no long-term reauthorization in place, stakeholders faced mounting concerns about the sustainability of U.S. global HIV/AIDS support amid shifting political priorities.

By early 2025, those concerns deepened, with Trump’s return to office and the imposition of a funding freeze. This shift reintroduced deeper structural instability, as the combination of past ideological restrictions, legislative gridlock, and executive funding cuts severely compromised the sustainability of HIV programs that were once models of global health success. These cumulative disruptions jeopardize the goals of the UNAIDS 95-95-95 targets, especially in low and middle-income countries that rely heavily on U.S. support for ART access and viral suppression. Since its launch in 2003, PEPFAR has been instrumental in the global HIV/AIDS response, aligning its goals with the UNAIDS 95-95-95 targets. The program has provided substantial support for HIV testing, treatment, and care services worldwide. It has focused on high-burden countries, particularly in Southern Africa. The cessation of U.S. funding is expected to have several detrimental effects and its impact is already being felt across the region with South Africa, expected to see over 500,000 additional HIV-related deaths in the next decade, and Ethiopia facing acute shortages of testing kits and delayed early infant diagnosis services due to procurement disruptions (40, 42). Without urgent alternative funding mechanisms, the gains made under PEPFAR are at risk of reversal, potentially placing millions at risk of increased mortality and a resurgence of the epidemic in affected regions.

This discussion underscores the persistent and multifaceted nature of barriers to ART adherence among adults living with HIV in sub-Saharan Africa. As the 2025 deadline approaches, current trends indicate that despite global commitment and years of strategic evolution, progress toward achieving the UNAIDS 95-95-95 targets remains uneven and insufficient. The findings reveal a complex matrix of individual, social, economic, and structural barriers, including mental health challenges, substance use, poverty, and fragile health systems, further exacerbated by the COVID-19 pandemic and the evolving HIV funding landscape.

While researchers and academic literature have yet to fully reflect the anticipated impact of 2025 HIV funding cuts, early signals from UNAIDS and other reputable sources predict an escalating situation that threatens to derail hard-won gains. As such, interventions must move beyond individual behavior change to multi-level, evidence-based strategies that integrate mental health support, social protection, and structural reform. The discussion has also highlighted the need to build resilient, community-based service models that can withstand funding volatility and health system dynamism.

Achieving and sustaining the 95-95-95 targets calls for a renewed commitment to addressing the root causes of non-adherence and ensuring that policies, funding mechanisms, and service delivery models are informed by the lived realities of the communities they are designed to serve.

## 5 Conclusion and recommendations

Achieving universal ART adherence remains a challenge due to economic, social, and individual-level barriers. This review has highlighted the need for multi-faceted interventions to improve adherence. mHealth tools, such as Call for Life Uganda (CFLU), have demonstrated effectiveness in medication reminders and appointment alerts; however, their design should be optimized for younger individuals and those with disclosure concerns. To improve adherence monitoring, integrating self-reports, pharmacy refill tracking, and point-of-care assessments is crucial, alongside strengthening clinic efficiency via flexible hours, reduced wait times, and decentralized services that could further support adherence.

Stigma remains a key barrier, which results in the fear of disclosure and treatment interruptions. Community-based adherence clubs and peer support groups can help normalize ART use and improve retention. Additionally, economic constraints such as job insecurity and transport costs continue to hinder adherence, particularly for men in informal employment and women balancing caregiving responsibilities. Financial incentives linked to clinic attendance have been shown to improve adherence by offsetting costs and income loss. Mental health is another critical factor, with depression, anxiety, and PTSD being strongly associated with poor adherence. Integrating mental health services into HIV care can enhance treatment retention. Behavioral interventions that reinforce habit formation, such as linking medication to daily routines and using reminders, can further improve adherence.

The evolving global HIV funding landscape also threatens ART access, in particular, the current cessation of USAID and PEPFAR allocations. Funding inconsistencies lead to the disruption of services, reduced access to ART, and the closure of clinics in some regions, further exacerbating adherence challenges. To mitigate the effects of shifting donor priorities, governments and stakeholders must prioritize domestic HIV/AIDS financing, strengthen local partnerships, and integrate HIV services into broader healthcare systems to ensure long-term sustainability.

In conclusion, ART adherence requires a multi-level response addressing structural, economic, social, and psychological barriers. The findings from this review highlight the need for integrated interventions, sustainable funding mechanisms, and tailored patient support strategies to improve ART adherence and advance progress toward the UNAIDS 95-95-95 targets. Future research should explore scalable, cost-effective interventions that enhance adherence while ensuring HIV care remains accessible and resilient to funding shifts. To achieve this, implementation science could guide the translation of evidence-based interventions into practice by identifying context-specific barriers and facilitators, optimizing delivery strategies, and evaluating real-world effectiveness and sustainability.

## 6 Limitations

- Possible exclusion of relevant studies published in languages other than English.
- Potential publication bias favoring studies from countries with the capacity to conduct research on ART adherence.
- This review only considers the barriers affecting adult men and women and does not include other vulnerable populations such as adolescents and the LGBTQ+ community, which have their unique challenges.
