# Supplementary material for: Barriers to ART adherence in sub-Saharan Africa: a scoping review toward achieving UNAIDS 95-95-95 targets
Source: Front Public Health. 2025 Jun 10;13:1609743. doi: 10.3389/fpubh.2025.1609743 (PMC12185539; doi:10.3389/fpubh.2025.1609743)
Supplement: Supplementary file 1 [file Table_1.docx]

| **Author** | **Title** | **Year & Country** | **Study Summary** | **Key Findings** | **Contributions** |
| --- | --- | --- | --- | --- | --- |
| **Saya et al** | “The one who doesn’t take ART medication has no wealth at all and no purpose on Earth” – a qualitative assessment of how HIV-positive adults in Uganda understand the health and wealth-related benefits of ART | 2022  Uganda | The study examines ART benefits for HIV-positive adults in Uganda. Participants perceive both the health and long-term economic benefits of ART.  Qualitative interviews were conducted with 40 individuals on long-term ART.  Findings highlight ART's impact on individual, household, and societal levels.  Improved health leads to increased productivity and social connections. Adherence challenges persist despite awareness of ART benefits.  Recommendations for interventions to enhance ART uptake and adherence | PLWH perceives both short and long-term benefits of ART adherence. Structural factors influence ART adherence and health outcomes. Findings can guide policymakers to improve HIV-related outcomes. Evidence suggests integrating structural benefits into ART interventions. | The study explores ART's health and wealth benefits for PLWH.  It highlights the long-term implications of ART beyond short-term health. Findings can guide policymakers in tailoring HIV care programs.  It provides qualitative insights into PLWH's perceptions of ART benefits. The research emphasizes the importance of ART adherence for productivity.  It addresses adherence issues among PLWHs in care settings. |
| **Twimukye et al** | Acceptability of a mobile phone support tool (Call for Life Uganda) for promoting adherence to antiretroviral therapy among young adults in a randomized controlled trial: Exploratory qualitative study | 2021  Uganda | The study evaluates a mobile health tool for ART adherence.  Young adults reported improved medication adherence using the CFLU tool.  Key benefits included appointment reminders and health tips.  Participants faced technical issues and stigma-related challenges.  Suggestions for improvement included diverse health information and support. | The CFLU tool improves medication adherence among young adults.  Participants reported better management of forgetfulness and stigma. Technical issues and financial constraints hindered CFLU usage.  Youth suggested improvements for the CFLU tool.  Four major themes emerged from participant interviews. | The study provides evidence on the effectiveness of CFLU tools. |
| **Belus et al** | Adapting a Behavioral Intervention for Alcohol Use and HIV Medication Adherence for Lay Counselor Delivery in Cape Town, South Africa: A Case Series | 2022  South Africa | The study addresses alcohol use and HIV treatment in South Africa. It presents the 'Khanya' intervention for lay counselor delivery.  The intervention combines counseling, motivational interviewing, and relapse prevention.  Case studies illustrate clinical adaptations and treatment delivery challenges.  Participants showed improved alcohol use and ART adherence.  The study emphasizes task sharing among healthcare providers.  Mindfulness techniques were beneficial for participants. | The study describes an integrated intervention for alcohol use and ART adherence. Common clinical challenges and strategies to overcome them were identified. Mindfulness skills were effectively taught to participants.  The intervention showed flexibility and required supervision for optimal delivery. | The intervention integrated evidence-based components for alcohol use and ART adherence. Participants understood clinical concepts from a different cultural context. The interventionist's understanding of mindfulness improved through personal practice. The study highlighted the need for ongoing training and supervision for lay providers. |
| **Jones et al** | Association between HIV stigma and antiretroviral therapy adherence among adults living with HIV: baseline findings from the HPTN 071 (PopART) trial in Zambia and South Africa | 2020  Zambia and South Africa | HIV stigma undermines ART adherence among people living with HIV. The study analyzed data from 21 communities in Zambia and South Africa. Poor adherence was reported by 15.8% of participants. Addressing stigma could improve lifelong ART adherence. | 15.8% of participants reported poor ART adherence.  Community stigma significantly affects ART adherence rates. Internalised stigma also correlates with poor ART adherence.  Health setting stigma shows a weak association with ART adherence. Higher alcohol consumption relates to increased internalised stigma.  Education level impacts ART adherence differently in countries.  Stigma increases food insecurity among individuals living with HIV. | The paper explores HIV stigma on ART adherence. It identifies community stigma as a significant adherence barrier.  The study provides evidence of stigma's association with poor adherence.  It highlights differences in stigma effects between Zambia and South Africa. The research informs strategies to improve ART adherence through stigma reduction. |
| **Wagner et al** | Changes in ART Adherence Relate to Changes in depression as Well! Evidence for the Bi-directional Longitudinal Relationship Between Depression and ART Adherence from a Prospective Study of HIV Clients in Uganda | 2020  Uganda | Depression and ART non-adherence are bidirectionally related over time. Changes in depression predict changes in non-adherence and vice versa. At baseline, 40.6% had minor depression; 37.1% were non-adherent. Depression treatment may improve adherence and mental health. | Increased non-adherence predicts increased depression. Time-varying changes in depression affect non-adherence status. Findings support the need for depression care and adherence support. | The study supports the bidirectional relationship between depression and non-adherence. Findings suggest depression treatment may improve ART adherence. The research highlights the need for further studies on mental health and adherence. It emphasizes the importance of addressing barriers to adherence and mental health. |
| **Mubiana-Mbewe et al** | Effect of Enhanced Adherence Package on Early ART Uptake Among HIV-Positive Pregnant Women in Zambia: An Individual Randomized Controlled Trial | 2021  Zambia | The study evaluated BEAP's effect on early ART uptake in Zambia.  Participants: HIV-positive pregnant women, randomized to BEAP or SOC.  98.2% initiated ART within 30 days of eligibility. BEAP showed higher adherence compared to standard care.  Early dropout indicates a need for additional adherence support. | BEAP improved ART uptake among option B+ women. 82.5% in BEAP and 80.4% in SOC reached the primary outcome. Early ART initiation was nearly universal, but dropout occurred. The study highlights the need for additional adherence support. | Development of the Enhanced Adherence Package (BEAP) intervention. Focus on improving Option B uptake among pregnant women.  Conducted formative research with various stakeholders. Evaluated early ART uptake in a randomized controlled trial.  Highlighted high initial ART uptake in Zambia.  Suggested further research on long-term outcomes and cost-effectiveness |
| **Fahey et al** | Financial incentives to promote retention in care and viral suppression in adults with HIV initiating antiretroviral therapy in Tanzania: a three-arm randomised controlled trial | 2020  Tanzania | Financial incentives improve retention and viral suppression in HIV treatment. The study was conducted in Tanzania, and 530 participants started ART. Results showed positive trends with varying incentive sizes. Further research is needed on the long-term effects of financial incentives | Small financial incentives improved retention in care and viral suppression.  86.1% achieved viral suppression with the larger incentive group. A positive trend was observed between incentive size and viral suppression.  The larger incentive group had higher retention in care at six months.  Financial incentives may alleviate poverty-related barriers to care.  Study supports implementing financial incentives in standard HIV care | Financial incentives promote HIV service utilization and adherence.  Incentives alleviate poverty-related barriers to care.  Small incentives provide motivational 'nudges' for treatment prioritization.  Study informs optimal implementation strategies for financial incentives. Evidence supports financial incentives for achieving viral suppression |
| **Belus, Joska et al** | Gender Moderates Results of a Randomized Clinical Trial for the Khanya Intervention for Substance Use and ART Adherence in HIV Care in South Africa | 2022  South Africa | The study examines gender effects on ART adherence and AOD use.  Khanya intervention showed different outcomes for men and women. Women had lower ART adherence compared to men in Khanya.  No gender differences were found for AOD outcomes. Further research is needed on gender-specific interventions | Gender moderated the effect of Khanya on ART adherence. Women in Khanya had lower ART adherence than men. No gender differences were found for AOD outcomes. Men showed significant reductions in AOD outcomes; women did not. | Men in the Western Cape show higher AOD use than women. Study examines gender differences in Khanya intervention outcomes.  Few studies address both AOD use and ART adherence.  Gender-specific barriers to treatment utilization need evaluation. Integrating gender into interventions can improve treatment outcomes |
| **Mayo-Wilson et al** | Habit formation in support of antiretroviral medication adherence in clinic-enrolled HIV-infected adults: A qualitative assessment using free-listing and unstructured interviewing in Kampala, Uganda | 2020  Uganda | ART adherence may decline despite initial motivation, risking health outcomes. Habits can support long-term ART adherence by becoming automatic practices. The study used free-listing and interviews with 42 clients in Uganda. Findings showed routine habits often lack consistent timing or location. Interventions targeting existing routines may enhance ART adherence | Clients' habits do not consistently align with ART pill-taking times. Tailoring interventions to client demographics may enhance ART adherence. Linking ART to established daily routines can strengthen adherence. Challenges include changing employment hours and social activities, which affect adherence. | The study examines ART adherence in treatment-mature adults. It qualitatively assesses habit formation for ART adherence.  The research identifies facilitators and barriers to habit formation.  It links ART medication to existing daily routines. |
| **Nakimuli-Mpungu et al** | Long-Term Effect of Group Support Psychotherapy on Depression and HIV Treatment Outcomes: Secondary Analysis of a Cluster Randomized Trial in Uganda | 2022  Uganda | Group support psychotherapy (GSP) improves depression and HIV treatment outcomes over 24 months. GSP participants showed lower depression rates compared to the group HIV education (GHE). Higher ART adherence and viral suppression were observed in GSP participants. The study highlights GSP's potential in rural, socially disadvantaged communities. | GSP participants showed higher ART adherence than GHE participants. Viral suppression rates were better in GSP than in GHE participants. Depression treatment with GSP is crucial for HIV treatment outcomes. GSP effects sustained over 24 months post-treatment.  Sequential changes in depression and ART adherence mediated viral suppression. | GSP showed significant long-term effects on depression and functioning. GSP may enhance ART persistence beyond improving adherence. GSP participants had lower depression rates compared to GHE at 24 months. GSP contributed to higher viral suppression rates than GHE |
| **Mcinziba et al** | Managing household income and antiretroviral therapy adherence among people living with HIV in a low-income setting: a qualitative data from the HPTN 071 (PopART) trial in South Africa | 2023  South Africa | Only 67% of PLHIV accessed ART as of 2021. Low-income households face barriers to HIV treatment adherence. The study explores income management and ART adherence interactions. Qualitative data from 21 PLHIV in the Western Cape were analyzed. | The study included 21 participants, aged 18-70, with diverse demographics. 15 participants reported adherence to ART, though some missed doses. Six participants struggled with consistent ART access and adherence. Household income management affected ART adherence experiences. | The study explores income management in HIV-affected households. It examines ART adherence influenced by household income stability. The research highlights barriers to accessing HIV treatment. It identifies diverse income management strategies among participants.  The study suggests creating a health-enabling environment for PLHIV. |
| **Conroy et al** | Mlambe economic and relationship-strengthening intervention for alcohol use decreases violence and improves relationship quality in couples living with HIV in Malawi | 2024  Malawi | Mlambe intervention improves relationship quality in HIV couples.  It reduces intimate partner violence (IPV) significantly.  Women benefit more from relationship improvements than men.  Economic and relationship interventions disrupt harmful syndemics.  Pilot study shows feasibility and acceptability of Mlambe. | Mlambe improved couple communication, unity, and sexual satisfaction significantly. Physical and emotional IPV decreased in the Mlambe arm compared to the control. Women reported greater relationship quality improvements than men. Rates of physical IPV declined from 25% to 18.5% over 15 months. Emotional IPV prevalence increased from 30.1% to 36.7% at follow-up | Mlambe intervention reduces unhealthy alcohol use and IPV in couples. It enhances relationship quality and communication among partners. The study highlights the couple interdependence theory in health behaviors. Findings suggest women report greater improvements in relationship quality. |
| **Keene et al** | Only twice a year: A qualitative exploration of 6-month antiretroviral treatment refills in adherence clubs for people living with HIV in Khayelitsha, South Africa | 2020  South Africa | Study explores 6-month ART refills in adherence clubs.  Participants included patients, healthcare workers, and key informants.  Six-month refills increased convenience and reduced clinic visits.  Patients were motivated and managed larger ART quantities well.  Strict eligibility criteria are necessary for extended refills.  Concerns exist about the drug supply chain's reliability.  Stepwise expansion is recommended to adapt health systems. | Six-month ART refills increased convenience for patients.  Patients were motivated and managed larger ART quantities effectively.  Strict eligibility criteria were deemed necessary for extended refills. Six-month refills improved health system efficiency. Concerns existed about the drug supply system's adaptability. Participants found six-month refills acceptable and beneficial.  Gradual expansion could prevent overstressing the supply chain | Patients found 6-month refills increased convenience and reduced unintended disclosure. Longer refills provided peace of mind and treatment management control. Six-month refills were seen as beneficial and acceptable by all participant groups. The study highlights the need for strict eligibility criteria for extended refills. Health system efficiency was perceived to improve with 6-month refills. |
| **Mudavanhu et al** | Perceptions of Community and Clinic-Based Adherence Clubs for Patients Stable on Antiretroviral Treatment: A Mixed Methods Study | 2020  South Africa | Adherence clubs improve care for stable ART patients.  Study compares community vs. clinic-based adherence clubs.  Participants favor clinic-based clubs over community-based clubs.  Concerns include stigma and limited access to healthcare services in community-based clubs.  A mixed-methods approach was utilized for data collection and analysis.  High acceptability ratings for both club types.  Clinic-based clubs are preferred for additional health services. | Participants favor clinic-based clubs over community-based clubs due to stigma concerns. Clubs promote social support and are time-saving for participants. Participants expressed concerns about confidentiality in community settings. | Adherence clubs improve clinic decongestion and task-shifting for ART patients. They enhance adherence and retention in care for stable ART patients. Participants rated adherence clubs favorably for convenience and social support. Concerns about stigma and healthcare access were noted in community-based clubs. |
| **Ngowi et al** | Predicting viral load suppression by self-reported adherence, pharmacy refill counts, and real-time medication monitoring among people living with HIV in Tanzania | 2022  Tanzania | The study evaluates adherence measures by predicting viral load suppression.  Self-report, pharmacy refill, and RTMM were assessed for sensitivity. Self-reported adherence had the lowest sensitivity and highest specificity. Combining all measures improved sensitivity, but it needs a feasibility study.  Pharmacy refill counts better predict virological failure than self-report. | Adherence measures did not predict virologic failure well individually. Combining self-report, pharmacy refill, and RTMM may improve predictions. Pharmacy refill adherence outperformed self-report in predicting virological failure. The study had a small sample size, suggesting further research is needed. The study compared three adherence measures in a randomized trial context. High self-reported adherence did not prevent virological failure. | The study compares adherence measures for predicting viral suppression. RTMM is less prone to overestimating adherence than self-report.  Self-reported adherence shows high specificity but low sensitivity.  Combining adherence measures improves sensitivity for predicting virological failure. |
| **Gumede et al** | Predictors of Treatment Adherence and Virological Failure Among People Living with HIV Receiving Antiretroviral Therapy in a South African Rural Community: A Sub-study of the ITREMA Randomised Clinical Trial | 2023  South Africa | Rural PLHIV face barriers to ART adherence and virological failure. Depression significantly impacts adherence and health outcomes in PLHIV. Socio-economic factors like income and food insecurity affect ART outcomes. | Male gender is a risk factor for adherence difficulties and virological failure. Food insecurity is linked to virological failure in males. Depressive symptoms are associated with virological failure in both genders. Task-oriented coping protects against suboptimal pill count adherence | The study identifies barriers to ART adherence in rural South Africa. It highlights the impact of socio-economic factors on treatment outcomes. Male gender is a risk factor for adherence difficulties and virological failure. Food insecurity and depression are linked to virological failure |
| **Magidson et al** | Project Khanya: a randomized, hybrid effectiveness-implementation trial of a peer-delivered behavioral intervention for ART adherence and substance use in Cape Town, South Africa | 2020  South Africa | Substance use in South Africa negatively impacts HIV treatment outcomes.  A task-shared intervention aims to improve ART adherence and reduce substance use. The trial evaluates a peer-delivered intervention using the RE-AIM framework. Implementation outcomes include feasibility, acceptability, and fidelity assessments. | The intervention improves ART adherence and reduces substance use. Peer delivery of the intervention is feasible and acceptable. Implementation outcomes include fidelity, acceptability, and feasibility assessments. Findings may inform the integration of behavioral health in primary care | The study evaluates a peer-delivered intervention for ART adherence and substance use. The research aims to improve HIV treatment outcomes in South Africa |
| **Campbell et al** | Stay-at-Home: The Impact of the COVID-19 Lockdown on Household Functioning and ART Adherence for People Living with HIV in Three Sub-districts of Cape Town, South Africa | 2022  South Africa | Lockdown affected ART adherence for people living with HIV in South Africa. Data was collected from 152 participants before lockdown; 83 followed up later. Increased poverty and decreased violence were noted during follow-up. Governments must ensure support and access to health services during lockdown. | Increased violence and poverty were reported during the lockdown period. Higher well-being scores correlated with better ART adherence. Further research is needed to evaluate long-term changes post-lockdown | The study highlights the impact of lockdown on ART adherence for PLWH. It identifies improvements in household functioning and stigma during the lockdown. The research emphasizes the importance of social support for well-being. It discusses the economic impact of the lockdown on household income |
| **Cassidy et al** | Twenty-four-month outcomes from a cluster-randomized controlled trial of extending antiretroviral therapy refills in ART adherence clubs | 2020  South Africa | The study compares standard care and six-month refill ART adherence clubs.  It reports non-inferior retention, VL completion, and suppression at 24 months. Higher VL completion and suppression were observed in the intervention arm. The findings support extended ART dispensing intervals for HIV patients | Six-month ART refills showed non-inferior retention in care.  Viral load completion was higher in the intervention arm.  Viral load suppression rates were also higher in the intervention arm.  Retention in care was over 92% for both arms.  Patients preferred fewer frequent visits and longer refills.  Study provides real-world evidence for extended ART dispensing intervals. | The study supports six-month ART refills for HIV patients.  It demonstrates non-inferior retention in care at 24 months.  Higher viral load completion and suppression rates were observed.  It adds to the literature on extended ART dispensing intervals. |
| **Katirayi et al** | Understanding gender differences of people with HIV newly diagnosed or returning to care with advanced HIV disease in Malawi: a qualitative study | 2023  Malawi | Study examines gender differences in HIV care decisions in Malawi.  Men delay HIV testing and ART initiation more than women.  Stigma significantly impacts both genders' decisions for HIV care.  Women are motivated by family health; men by personal health.  Treatment fatigue was reported as a reason for discontinuing ART.  Gender-tailored counseling services are recommended for better outcomes. | Gender differences affect decisions for HIV testing and ART adherence. Stigma is a significant barrier to HIV testing and treatment. Men tend to test later for HIV than women. Treatment fatigue is a common reason for discontinuing ART. Motivations for accessing HIV care differ between genders. | The study highlights gender differences in HIV care decisions.  It identifies stigma as a barrier to HIV testing and treatment. The research emphasizes the need for gender-tailored counseling services.  It explores logistical challenges faced by men and women.  The findings inform public health interventions for better health outcomes. |
| **Camlin et al** | Understanding the role of incentives for achieving and sustaining viral suppression: A qualitative sub-study of a financial incentives trial in Uganda | 2022 Uganda | Financial incentives may improve ART adherence among PLHIV.  Mixed results were observed in achieving viral suppression with incentives.  Barriers include transport costs, food insecurity, and stigma.  Gender differences affect care engagement and adherence.  Incentives alone may not overcome structural barriers.  Combining incentives with other interventions is recommended. | Financial incentives motivated ART adherence among some participants.  Incentives reduced transport costs and food insecurity burdens.  Mixed results were observed in achieving viral suppression with incentives.  Subgroup analyses indicated that unsuppressed participants did not respond to incentives.  Barriers included food insecurity and poor treatment experiences in clinics. Health systems interventions are needed for effective care engagement. | Financial incentives may improve ART adherence and reduce transport costs. Incentives alone are insufficient to overcome multiple barriers to care. Combining incentives with other interventions may enhance viral suppression outcomes. Supportive counseling and quality care are essential for patient engagement |

**Abbreviations**: ART – Antiretroviral Therapy; PLWH – People Living with HIV; CFLU – Call for Life Uganda; AOD – Alcohol and Other Drugs; BEAP – Better Early ART Package; GSP – Group Support Psychotherapy; GHE – Group HIV Education; RTMM – Real-Time Medication Monitoring; VL – Viral Load; IPV – Intimate Partner Violence; RE-AIM – Reach, Effectiveness, Adoption, Implementation, and Maintenance; SOC – Standard of Care; PLHIV – People Living with HIV.
